# Diet Alters Both the Structure and Taxonomy of the Ovine Gut Microbial Ecosystem

**DOI:** 10.1093/dnares/dst044

**Published:** 2013-10-29

**Authors:** Melinda J. Ellison, Gavin C. Conant, Rebecca R. Cockrum, Kathy J. Austin, Huan Truong, Michela Becchi, William R. Lamberson, Kristi M. Cammack

**Affiliations:** 1 Department of Animal Science, University of Wyoming, Laramie, WY, USA; 2 Division of Animal Sciences, University of Missouri, Columbia, MO, USA; 3 Informatics Institute, University of Missouri, Columbia, MO, USA; 4 Department of Animal Science, Colorado State University, Fort Collins, CO, USA; 5 Department of Electrical and Computer Engineering, University of Missouri, Columbia, MO, USA

**Keywords:** *Ovis aries*, microbiome, 16S subunit

## Abstract

We surveyed the ruminal metagenomes of 16 sheep under two different diets using Illumina pair-end DNA sequencing of raw microbial DNA extracted from rumen samples. The resulting sequence data were bioinformatically mapped to known prokaryotic 16S rDNA sequences to identify the taxa present in the samples and then analysed for the presence of potentially new taxa. Strikingly, the majority of the microbial individuals found did not map to known taxa from 16S sequence databases. We used a novel statistical modelling approach to compare the taxonomic distributions between animals fed a forage-based diet and those fed concentrated grains. With this model, we found significant differences between the two groups both in the dominant taxa present in the rumen and in the overall shape of the taxa abundance curves. In general, forage-fed animals have a more diverse microbial ecosystem, whereas the concentrate-fed animals have ruminal systems more heavily dominated by a few taxa. As expected, organisms from methanogenic groups are more prevalent in forage-fed animals. Finally, all of these differences appear to be grounded in an underlying common input of new microbial individuals into the rumen environment, with common organisms from one feed group being present in the other, but at much lower abundance.

## 1. Introduction

Microbial symbionts of mammals are ubiquitous, taxonomically diverse and highly abundant.^1^ Moreover, the word symbiont is used advisedly: among their many roles, gut microbes are critical in extracting nutrition for their hosts from the varied mammalian diets,^2^ with both diet and host phylogeny being necessary predictors for understanding gut microbe diversity.^3,4^ The complement of these organisms varies by individual within a species,^5^ and this variation can alter host phenotypes,^6,7^ a fact that makes understanding the diversity and function of these organisms of more than ecological interest.

Although other techniques are becoming available,^8^ the majority of current metagenomic studies have employed the sequence of the 16S subunit of the prokaryotic ribosome for taxa identification.^9^ This gene is appealing as it should be universal and permits the use of generic PCR primers that allow amplification from very diverse taxa in a single thermocycler reaction.^1^ As such, sequencing of 16S genes avoids the very serious biases inherent in any approach to microbial diversity that requires culturing.^1,9–12^ More recently, it has become possible to shotgun sequence raw metagenomic samples at high depth,^8,13^ presumably avoiding the potential for PCR-based artefacts that can occur when directly amplifying the 16S gene^14^ and allowing researchers to more fully explore the genic diversity of this ecosystem.

Such ecosystem probing may be especially rewarding when studying ruminants, because they are particularly dependent on their gut microbial symbionts. The reason for this dependence is that the cellulose and other plant materials that form the basis of their diets cannot be degraded by enzymes encoded in their own genomes.^2^ Instead, many different microbial taxa^9,15^ are responsible for producing a variety of enzymes that break down these plant cell components.^8,13^ Thus, in addition to the health-related concerns seen in microbiome studies in humans,^5^ understanding the microbiome of domestic animals has ecological and economic relevance.

The complement of microbes in the rumen can alter several host phenotypes: both the overall microbe composition and the distribution of methanogenic microbes differ between cattle with high efficiency of converting ingested food into biomass and those with lesser efficiency.^15,16^ The precise nature of the animal's diet also directly influences the gut microbiota. In cattle, there are clear differences in the relative abundances of different microbial taxa (hereafter microbial distributions), depending on the type of grass consumed.^17,18^

Here, we sought to better understand how diet alters rumen microbial diversity, using a shotgun sequencing approach that allowed deep sampling of microbial diversity across multiple individuals. Our goal was to understand the structural differences between two ecosystems, each defined by the host diet.

## 2. Methods

### 2.1. Animal trial and DNA sample collection

Growing wethers (*n* = 77; initial body weight = 51.3 ± 1.2 kg) of Rambouillet, Hampshire, and Suffolk breed types were randomly allocated by body weight to receive either a concentrate- (*CONCEN*: 50% corn, 31% wheat middlings, yielding a measured dietary intake of 91.6% dry matter including 12.1% crude protein, 17.6% neutral detergent fibre, and a mean energy of 2.98 Mcal/kg; *n* = 39 animals) or forage-based (*FORG*: 67.7% alfalfa, 27.5% wheat middlings, yielding a measured dietary intake of 92.3% dry matter including 16.2% crude protein, 36.3% neutral detergent fibre, and a mean energy of 2.31 Mcal/kg; *n* = 38 animals) pelleted diet. Lambs were acclimated to diets using a 20% increase in the proportion of new-to-old feed every 4–5 days until the diet consisted of 100% new pelleted diet *ad libitum*. To give the clearest sense of the microbial diversity across these two diets, individuals were selected for metagenomic sequencing based on their rate of body weight gain relative to feed intake. To do so, individual feed intake was measured using the GrowSafe System for a 49-day trial period. Two-day average initial and final body weights were obtained to calculate daily gain. We used residual feed intake (RFI) in order to select 16 animals for metagenomic sequencing. Thus, RFI was calculated as the deviation of true feed intake from expected feed intake. Expected feed intake was determined by regressing daily gain and metabolic midweight on actual feed intake.^19^ RFI calculations were used to rank wether efficiency. Rumen fluid samples were collected at the end of the feeding trial and frozen at −80°C. DNA was then extracted from the fluid of the 10% most (*n* = 4, low RFI) and the 10% least (*n* = 4, high RFI) efficient wethers from each diet (eight animals per diet, *n* = 16).

### 2.2. DNA extraction and library preparation

Sterilized zirconia (0.3 g of 0.1 mm) and silicon (0.1 g of 0.5 mm) beads and 1 ml of lysis buffer were added to thawed rumen fluid samples, and tubes were homogenized using a Mini-Beadbeater-8 at maximum speed for 3 min, incubated at 70°C for 15 min with gentle mixing every 5 min, and centrifuged at 4°C for 5 min. Supernatant was transferred into new 2-ml flat cap tubes and fresh lysis buffer was added to the pelleted beads. The homogenization, incubation, and centrifugation were repeated, and the supernatants were pooled. Precipitation of nucleic acids, removal of RNA and proteins, and purification were completed using the protocol of the QIAamp DNA Stool Mini Kit (Qiagen, Santa Clarita, CA, USA). Genomic libraries from these 16 samples were constructed following the manufacturer's recommended protocol with reagents supplied in Illumina's DNA sample preparation kit. Briefly, genomic DNA was sheared using standard Diagenode BioRuptor methods to generate fragment sizes of ∼300 bp. The resulting 3′ and 5′ overhangs were removed by an end-repair reaction that uses a 3′- to 5′-exonuclease activity and polymerase activity to blunt the fragment ends. A single adenosine nucleotide was added to the 3′ ends of the blunt fragment followed by the ligation of Illumina adapters. The resulting adapter-ligated fragments were size selected on an agarose gel. Fragments of ∼420 bp were excised from the gel and recovered from the gel slice by elution and ethanol precipitation as described by the Illumina protocol. Each purified library was quantified with a Qubit assay and library fragment size confirmed by the Agilent BioAnalyzer High Sensitivity DNA assay.

### 2.3. Metagenomic sequencing, quality filtering, and identification of novel 16S genes

Libraries were diluted and sequenced according to Illumina's standard sequencing protocol on a HiSeq 2000. The 16 libraries were multiplexed four libraries per lane, resulting in 100 bp, paired-end sequences. The mean insert size across the 16 samples was 309 bp, corresponding to an unsequenced insert between reads of ∼109 bp. Raw sequence reads are available from NCBI's short read archive (Project SRP028527).

Paired-end reads were quality filtered by truncating each read after the first run of three bases, with a phred quality score of <15.^20^ From the filtered reads, any read pair where one or both reads were <85 bases long or had an average quality score of <25 was omitted. The resulting reads represent 96 gigabases of sequence.

We then used the software package EMIRGE^21^ to identify potentially unknown 16S rDNA sequences in these data. EMIRGE uses a reference 16S database (see below) and the Bowtie alignment tool^22^ to identify sequence reads that are potentially derived from 16S rDNA genes. It then iteratively constructs a set of new consensus 16S sequences found in the metagenomic sample, but not in the reference database.

### 2.4. Classification of 16S rDNA-derived reads

To identify reads derived from 16S rDNA genes, we compared the filtered reads to two distinct reference databases of 16S rDNA genes. The first database (16S_Ref) was constructed by combining the Ribosomal Database collection of sequences^23^ and the set of 16S rDNA genes from the sequenced prokaryotic genomes at NCBI GenBank.^24^ Identical sequences were purged from the database, as were sequences of <1450 bases long and those with undetermined nucleotides (e.g. ‘N's), resulting in a final database of 27 290 sequences. The second database (16S_Merge) comprised the union of 16S_Ref and the novel 16S sequences identified above with EMIRGE. We then used Bowtie^22^ to align reads from our 16 animals to these two databases. For both the forward and reverse reads, we required at least 97% sequence identity between the read and the database sequences. We retained both the best hit for each read and a second list of all database sequences, where both members of a read pair aligned with a ≥97% sequence identity. This second list was retained in order to perform the sequence clustering and operational taxonomic unit (OTU) identification described below. In Table 1, we list the number of identified bacterial individuals in each sample that met these criteria.

**Table 1.** Mapping Illumina reads to 16S rDNA databases and OTU identification

Sample	Diet	Million paired reads^a^	Individual 16S genes^b^	% of reads from 16S^c^	*P*^d^	Total OTUs^e^
1003	*FORG*	16.8	860/2718	0.016		109/419
1009		35.9	2548/8935	0.025		161/489
1127		41.1	1744/5229	0.013		137/467
1208		44.8	2731/11 431	0.026		140/539
1248		22.7	2078/5232	0.023		127/470
1366		18.1	1615/3264	0.018		119/440
1397		32.2	2184/5327	0.017		137/491
7505		47.2	3049/7571	0.016		177/510
Total		258.9	16 809/49 707	0.019	<10^−10^	280/801
1026	*CONCEN*	29.8	6174/22 787	0.076		108/225
1101		54.9	6401/13 579	0.024		142/297
1111		26.7	2904/18 633	0.070		137/289
1220		7.8	929/3758	0.048		75/172
1239		42.2	5296/19 310	0.046		138/276
1348		13.6	1825/5055	0.037		102/222
1396		18.3	1996/8497	0.046		124/289
7429		30.2	3745/12 735	0.042		135/345
Total		223.6	29 270/104 354	0.047		250/574
Grand total		482.5	46 079/154 061	0.032		349/992

^a^Total number of paired reads (over 1 million) analysed after quality filtering.

^b^Number of paired reads that both mapped onto at least one 16S gene in the database with a >97% identity. A/B # of reads mapped onto 16S_Ref /16S_Merge (Methods).

^c^% of reads identifiable as 16S genes when both database and EMIRGE sequences are considered.

^d^*P*-value for the hypothesis test that the proportion of mappable 16S reads was the same for the forage and concentrate diets (for both databases 16S_Ref and 16S_Merge; see Results).

^e^Number of distinct OTUs observed for each sample: A/B: # of OTUs when considering 16S_Ref versus when considering 16S_Merge (Methods).

There were 8472 and 9188 gene sequences from 16S_Ref and 16S_Merge found to match our reads, respectively. In each case, we performed single-linkage clustering using custom software. To do so, we first computed all possible pairwise global alignments between the genes using our new GPU-based global pairwise alignment package.^25,26^ We next created a graph where each node was a 16S rDNA gene. We defined edges between pairs of genes if their pairwise global sequence identity was ≥97%.^27^ We then defined the OTUs to be the connected components in this graph.

Using in-house perl scripts, we mapped these OTUs back onto the reads, using each read pair's top hit to assign that pair to an OTU. We identified 349 OTUs using 16S_Ref and 992 OTUs with 16S_Merge (Table 1). To test whether the percentage of reads mapped onto the rDNA database was the same for the two feed groups, we fit the number of reads mapped over the total number of reads to a binomial distribution, first requiring that proportion of reads mapped (*p*) be the same for both groups, then allowing *p* to differ between diets. Twice the difference in ln-likelihood for these models was compared with a *χ*^2^ distribution with one degree of freedom (e.g. a likelihood ratio test).^28^

### 2.5. Phylum-level analysis

Using the taxonomic names from the 16S_Ref database, we analysed the phyla-level distribution of our OTUs, mapping each prokaryotic taxa or genus name to the NCBI taxonomy database^29^ to retrieve the corresponding phylum.

### 2.6. Statistical comparison of metagenome populations between individuals differing in diet

The metagenomic sequence data collected here are unusual in that similar environments have been sampled multiple times (e.g. sheep fed the same diet). We therefore require computational and statistical approaches able to statistically assess if the two diets induce a difference in microbe distribution. To detect any significant differences in microbial taxa (OTU) abundance between the animals with different feeds, we developed a partial statistical model, implemented in custom c++ programs. The input data for this model are the raw counts of OTU observations from each animal (Table 1). However, because different numbers of total microbial individuals were sequenced for each animal host, it is not appropriate to directly compare these counts. Instead, the model is based on the underlying assumption that the relative abundances of the different OTUs in the rumen follow a multinomial distribution. In other words, OTUs *i* = 1…*n* each have a relative frequency *p_i_* in the environment such that:

(1) $$\sum\limits_{i = 1}^n {\,p_{\rm i} = 1} $$

These *p_i_s* then give the probability that a single microbial individual drawn from that animal would come from OTU *i*. The probability of the observed bacterial OTU counts from an animal *j* (*D_j_*) is then given by:

(2) $$P(D_j ) = \left( {\displaystyle{{n\hbox{!}} \over {\sum\limits_{i = 1}^n {x_i \hbox{!}} }}} \right) \cdot \prod\limits_{i = 1}^n {\,p_i^{x_i } } $$

where the *x_i_s* give the number of individuals observed from OTU *i*. The obvious difficulty with this model is that it has *n*− 1 unknown parameters (the *p_i_s*). With a sample of only 16 individuals, estimating so many unknowns is infeasible. Instead, we assumed that the rank-ordered values of the *p_i_s* followed one of two discrete probability distributions: a discrete power-law or a geometric distribution (for discussion of this assumption, see McGill *et al*.^30^, and Izsák and Pavoine^31^). Thus, we took the total number of microbial individuals from each OTU across all animals and sorted this sum across all OTUs. We then defined *p*_1_ as the proportion of all microbial individuals that belonged to the most abundant OTU, *p*_2_ as the proportion belonging to the next most abundant and so forth. In this framework, the two probability distributions define the relationships between *p*_1_, *p*_2_, *…*, *p_n_*. Specifically, for the power-law distribution, the value of *p_i_* for the *i*th most abundant OTU (across all animals) is given by:

(3) $$p_i = \displaystyle{{i^{ - a} } \over {\sum\limits_{\,j = 1}^\infty {\,j^{ - a} } }}$$

where *a* is a parameter estimated from data (see below). Similarly, under the geometric distribution, the *p_i_* for the *i*th most abundant OTU is:

(4) $$p_i = \pi \cdot (1 - \pi )^{i - 1} $$

Where *π* is a parameter to be estimated. Thus, in both cases, we have reduced the problem from estimating *n* − 1 parameters to estimating one parameter. To do so, we fit the observed OTU counts to these models by maximum-likelihood. The likelihood of an entire sample of animals *L* is then given by the product of the *D_j_s* from (2). We estimate *a* or *π* using numerical optimization to find the value that maximizes *L.* ^32^

Now that the data have been placed into a modelling framework, we can use the models to ask if different samples follow different multinomial distributions. To test for differences between the samples due to diet, we adopted a partitioning and randomization approach. First, we divided the OTU distributions into the two dietary groups: *FORG* and *CONCEN* described above. We then individually calculated ln(*L*_F_) for *FORG* and ln(*L*_C_) for *CONCEN* and computed *D* *=* [ln(*L*_F_) + ln(*L*_C_)]*–*ln(*L*). Note that *FORG* and *CONCEN* differ from the full dataset potentially in both the rankings of the 349 or 992 OTUs and the value of *a* or *π*. Thus, *D* is a measure of how much samples *FORG* and *CONCEN* differ. To assess if the observed difference would be expected by chance, we randomly repartitioned the full dataset *A* into samples of the same size as *CONCEN* and *FORG* 1000 times. For each such randomization, we calculated the value of *D*_rand_. If *D* for the real dataset is exceeded by not more than 5% of the values of *D*_rand_, we can statistically conclude that there is sufficient evidence to reject the null hypothesis of the same species distribution in *CONCEN* and *FORG*.

### 2.7. Identifying OTU-level differences between feeds

The above approach only indicates whether or not the two feed groups are statistically distinguishable. It cannot describe the particular OTUs that drive this difference. In order to do so, we slightly modified our model to consist of three distinct multinomial distributions of the form of (1): *M*_S_, *M*_F_, and *M*_C_. Each distribution has its own value of *a* or *π.* Among the *n* OTUs, each can either be assigned to the shared distribution (*M*_S_) or to the distinct distributions (*M*_F_ and *M*_C_): this assignment is coded as a binary vector $\overline S $ of length *n*. The likelihood of a sample is then the product of the likelihood of the shared OTUs (*s_i_* = 0) under *M*_S_ and the distinct OTUs (*s_i_* = 1) under either *M*_F_ or *M*_C_, depending on the feed treatment for that sample. There are 2*^n^* possible values of $\overline S $, and we used our previously described simulated annealing software to search for the combination of the entries of$\overline S $ and the values of the three *a*’s or *π*'s that give the maximum likelihood of observing the data collected.^33^ We also compared the proportion of individuals who were members of the Methanobacteria group between the two feeds using the same binomial model used to test the read-mapping proportion.

## 3. Results

Using Illumina sequencing, we obtained >480 million paired-end reads from the rumen metagenomes of 16 sheep. We used two strategies for analysing the microbial taxonomic diversity present in these animals. First, by mapping the reads to known 16S rDNA genes (16S_Ref, Methods), we identified 349 known prokaryotic OTUs present in at least one of our 16 animals (Methods; Table 1). Secondly, by using the EMIRGE package,^21^ we assembled probabilistic consensus sequences for new 16S rDNA genes (16S_Merge), resulting in between a 2- and 4-fold increase in the number of reads identified as coming from 16S rDNA genes and roughly a 3-fold increase in the number of OTUs seen (Table 1).

In keeping with EMIRGE's described function of identifying new 16S rDNA sequences, <2% of the OTUs derived from EMIRGE 16S rDNA assemblies also included sequences from the existing database, strongly suggesting the presence of many unknown taxa in these samples.

**Figure 1.**
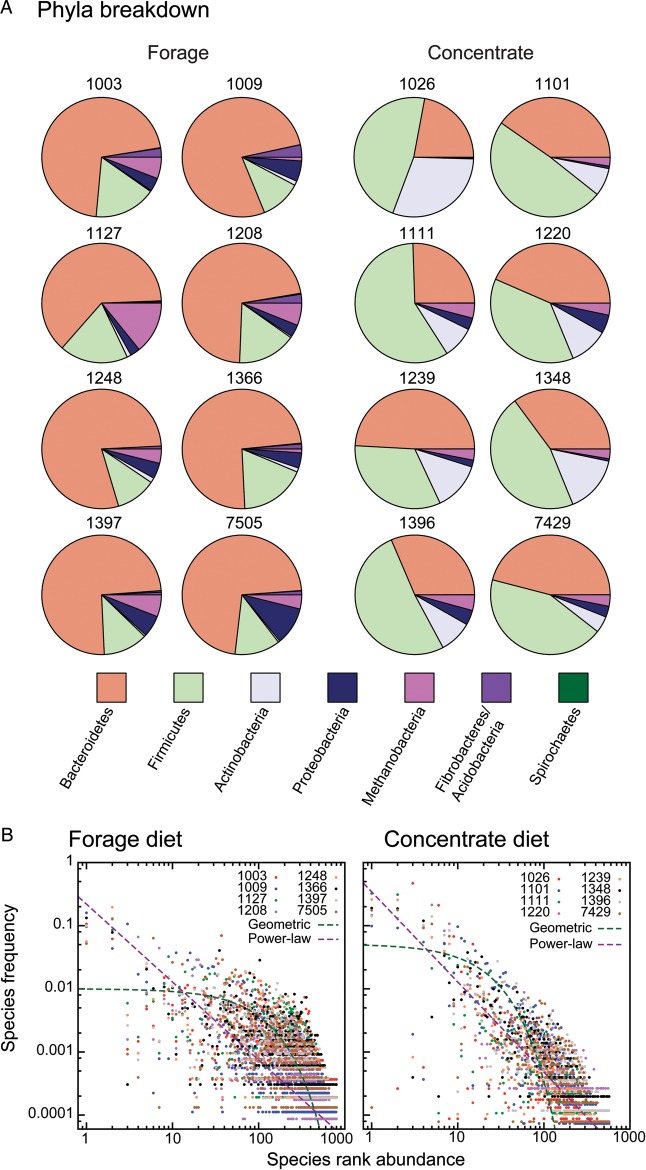
Microbial diversity in forage- and concentrate-fed animals. (A) Phylum-level breakdown of the microbial diversity, showing the top seven detected phyla for genes drawn from the 16S_Ref database (Methods). While there is considerable variation among individuals, there are clear differences between the two diets. Because all archaeans seen were from the Class Methanobacteria, this name is indicated. (B) Models of the species abundance curves for the forage diet (*FORG*), including all OTUs (e.g. 16S_Merge; Methods). On the *x*-axis is the rank abundance of each OTU: the most abundant OTU is rank 1 and so forth. On the *y*-axis is the proportion of the total sample for that individual that rank makes up. We fit two statistical distributions to these data: a discrete power-law (purple) and a geometric (green; Methods). For this diet, the geometric distribution provides a better fit (ln-likelihood of −278 825 versus −284 582 for the power-law distribution). (C) As in (B), but for the concentrate-fed animals (*CONCEN*). Here, the power-law distribution is a better statistical fit (ln-likelihood of −416 927 and −380 366 for the geometric and power-law distributions, respectively).

When considering gross, phylum-level differences between the animals in known taxa (16S_Ref), there is a clear distinction between the two feed conditions (Fig. 1A). Interestingly, the proportion of Illumina reads mapped onto 16S_Merge was roughly 2-fold higher among the concentrate-fed animals (1 in 2100 versus 1 in 5200), a significant difference (*P* < 10^−10^, likelihood ratio test, Methods). This bias is not attributable to an overall lower efficiency in obtaining DNA from these animals, as the raw number of reads obtained for each group is comparable (Table 1).

Methane production is a topic of considerable current interest,^34^ and consequently, we sought to assess if the abundance of methanogenic microbes differed between the two diet groups. As can be seen from Fig. 1A, there is considerable variation in the proportion of archaeans among the samples. All of these individuals were derived from one class among the Euryarchaeota, namely the Methanobacteria: they are indicated in pink in Fig. 1A. Nevertheless, on average, there are significantly more such microbes in animals administered a forage diet (*P* < 10^−10^; likelihood ratio test), a fact potentially related to the lowering of rumen pH under concentrate-type diets.^35^

To explore these differences in a rigorous statistical manner, we examined the relative abundance differences between samples (Fig. 1B and C). To assess whether there were systematic differences in the OTU abundances depending on feed source, we fit maximum-likelihood models of species abundances to our 16 samples under both an assumed power-law and geometric distribution of rank abundances (Methods). We first asked if the animals fed concentrate diets showed differing OTU distributions than did those fed forage diets. For both the power-law and geometric models, there was a significant improvement in fit by allowing the two feed groups to have their own multinomial distributions (*P* < 0.001 using either 16S_Ref or 16S_Merge).

**Figure 2.**
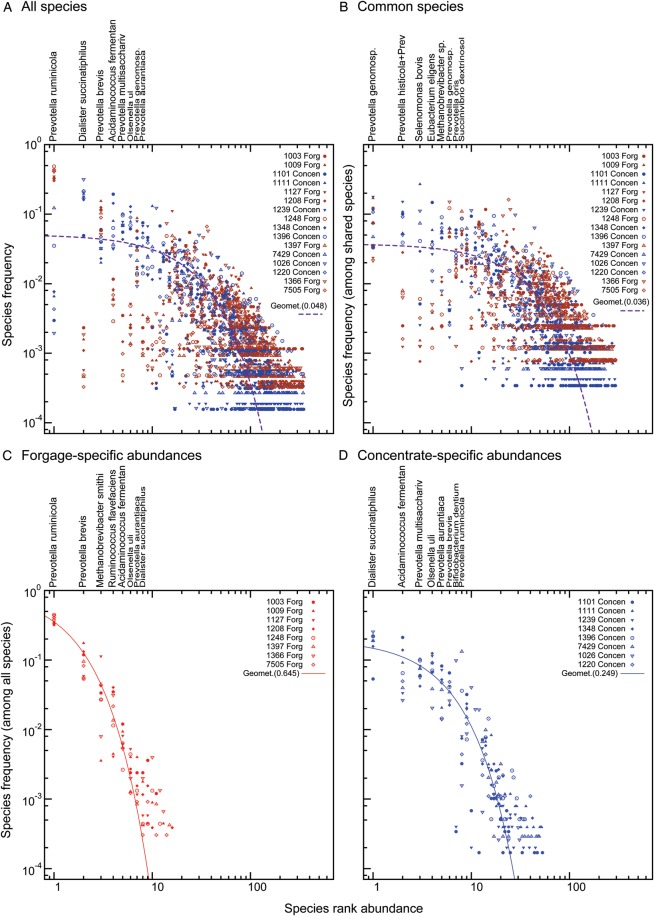
Distinct sets of high-abundance taxa between forage- and concentrate-fed animals are overlaid on a common core of rare organisms. For each panel, the *x*-axis gives the rank of each OTU (according the scheme for that panel), whereas the *y*-axis is the frequency of that OTU in a particular animal. Unlike Fig. 1B, here only genes matching to 16S_Ref are included (Methods). (A) The OTU distribution seen when all animals' OTU frequencies are plotted against the average OTU abundance across all 16 animals. The predicted abundance curves from our power-law and geometric distributions provide a visually very poor fit to the data, with obvious differences in abundance between the two feed groups (red, forage and blue, concentrate). (B–D) A machine-learning approach was applied to partition the set of 349 OTUs into either a ‘shared’ group common to both feeds or a feed-specific group (Methods). Generally speaking, this approach placed the abundant taxa into feed-specific groups (C: forage-feed animals, *FORG*; D: concentrate-fed animals, *CONCEN*), while there was a set of low-abundance microbes that did not appear to differ between the feeds. Thus, in C and D, the OTUs are individually ranked for the forage- and concentrate-fed animals, while in B, both groups share a common ranking. Note that, unlike Fig. 1, this partitioning approach yields curves that visually match the geometric distribution well. Representative taxa names are given above abundant organisms for reference.

This observed improvement in fit could result from a range of circumstances, from a large difference in abundance for a few OTUs to nearly non-overlapping OTU for the two treatments. Therefore, to understand the source of these differences, we applied a partitioning model that broke the OTUs down into two groups, one for which abundance was similar in both treatments and one for which each treatment had an independent abundance rank for that OTU (Methods). This approach is most appropriate when the OTUs analysed can be mapped to known taxa, and so we applied it to the OTUs found with 16S_Ref. We sought the maximum-likelihood arrangement of OTUs into these two groups. The two treatments are generally different in their most abundant OTUs (Fig. 2C and D: c.f. to A), with a group of more rarely observed OTUs with similar (low) abundances between the two treatments (Fig. 2B).

## 4. Discussion

### 4.1. High diversity in sheep ruminal metagenomes, with strong distinctions due to diet

We highlight two key findings from our analyses of rumen metagenomic DNA from sheep. First, there is evidence for a large number of currently unclassified microbes in this environment. EMIRGE predicted a number of new 16S rDNA sequences that do not cluster with existing sequences in the 16S database, and these sequences represent the majority of the 16S rDNA reads identified. Secondly, there are large differences in microbial distributions between the two diets examined, regardless of the 16S database used (16S_Merge, Fig. 1B and C and 16S_Ref, Supplementary Fig. S1).

### 4.2. Comparing microbial diversity across individual animals

Many discussions of the rumen microbial community quantify the complexity of the microbial community in terms of the number of species or OTUs.^9,14,17,18^ Here, we have chosen not to use that metric for several reasons. First, and trivially, the highly skewed distributions of the form of Fig. 1 suggest that while there may be a large number of lowly abundant taxa, it seems unreasonable to believe that the major differences between animals or diets result from these rare individuals. Secondly, most communities are described by two inter-related parameters, the richness (related to the number of taxa present) and the evenness (describing those taxa's relative abundance). Species abundance curves link these two concepts with a probability distribution, allowing fair comparisons between samples.^36^ Finally, we believe that the methods used to define OTUs in metagenomic contexts are unstable relative to sample size. We, like other researchers, have defined OTUs based on a 97% or greater sequence identity in the 16S rDNA gene. While this approach is sensible, it rests on an implicit network clustering approach whereby sequences are first linked by sequence identity, followed by a clustering step that defines connected components in a graph and hence OTUs (see Methods). However, adding sequences increases the chance of a new sequence bridging two previously separate OTUs. Thus, we expect that larger samples, while increasing the OTU count with new taxa, will also tend to compress that count through OTU merging. This effect is unlikely to have serious consequences in most cases, but it does mean that the OTU counts for different studies should not be directly compared. Our results are also unusual in that, because of our Illumina-based approach, we clustered not the sequence data but rather the ∼9000 database sequences that those reads matched to (Methods). As a result, our OTU estimates should not be compared with PCR-based analyses.^17,18^

### 4.3. Caveats

Our Illumina sequencing-based approach has different biases than do culture or PCR-based methods. Our read-mapping strategy precludes the identification of taxa with 16S genes <97% identical to known samples. This limitation is likely the reason that, although we had similar numbers of sequence reads for the two diets, the number of identified 16S genes was lower in the forage-fed group (Table 1). Likewise, because we did not sequence entire 16S genes, it is possible that certain OTUs might contain individuals who, while having 97% identity in some regions of the gene, are more dissimilar in other regions. Fortunately, this bias is constant across our samples. Another issue with all 16S-based approaches is that 16S copy number is taken as a proxy for microbe abundance, even though 16S copy number is not constant across genomes. Again, this effect should not bias our analyses, because it influences them all equally. Finally, the EMRIGE approach, while powerful, has a few shortcomings. First, the sequences inferred do not necessarily represent particular microbes from the sample, but are rather consensus inferences. It is therefore potentially dangerous to try to place them in a phylogenetic context. Moreover, the EMIRGE pipeline requires known 16S rDNA sequences as input: there still may be highly diverged 16S rDNA that have been missed.

Our results differ in detail somewhat from a previous analysis of the bacterial composition of both forage and concentrate-fed sheep that focused on the genus *Prevotella.*^37^ These authors found a higher percentage of *Prevotella* individuals in concentrate-fed animals, in contrast to our results finding that *Prevotella* was the dominant genus in forage-, but not concentrate-, fed animals (Fig. 2). Given the very different methods employed, it is difficult to know what to make of this difference. While the majority of the *Prevotella* found in an earlier bovine survey were from taxa not in 16S_Ref,^38^ they are unlikely to represent the most common OTUs here, since none of the five most abundant 16S rDNA sequences produced by EMIRGE had *Prevotella* as the strongest BLAST hit (data not shown). We note, however, that the general conclusion in both cases was that there was a greater diversity in the forage group.^37^

### 4.4. Diet-based differences in highly abundant microbes derived from a common core of taxa

As an alternative to the OTU counting approach mentioned above, we have described microbial diversity in terms of simple mathematical models (Figs 1 and 2). One apparent trend is the presence of a universal rare ‘core’ of organisms present in both groups (Fig. 2B). It is possible that this core is the result insufficient statistical power in our model. However, inspection of Fig. 2 shows some taxa with clear separation between the feeds (e.g. *Prevotella ruminicola* and *Dialister succinatiphilus* in Fig. 2A) and others with overlapping distributions (e.g. *Selenomonas bovis* in Fig. 2B). Instead, we suggest that another possibility is that a relatively large number of new microbial individuals enter the rumen, a suggestion supported by the observation that there are almost no OTUs of high abundance in one animal that are not at least found occasionally in all the other animals. Indeed, in only two microbial groups (*Parascardovia denticolens* and *Allisonella histaminiformans*) were 100 or more microbial individuals present in one feed group, with no individuals being present in the other. Thus, under this common inputs hypothesis, the observed differences are not a result of differences in microbes entering the system, but rather in the niches available to them when they arrive.

In support of this idea of reasonably high microbe turnover is the fact that the two diets differ not only in the OTUs present, but also in the nature of the taxa abundance curves. When the diets are treated separately (Fig. 1) and all 16S rDNA sequences are used, the microbial ecosystem induced by the forage diet is clearly more diverse than that induced by the concentrate diet (a ‘flatter’ power-law curve in Fig. 1B for the forage diet versus Fig. 1C and the concentrate-fed animals). This result may appear to contradict the data of Fig. 2C and D, where the forage diet has a rumen community that is dominated by a single OTU (*P. ruminicola*). However, we believe that this apparent discrepancy results from the fact that the reference database used in that figure (e.g. 16S_Ref) more poorly represents the highest abundance taxa from the forage environment than from the concentrate-induced one. Thus, the slope seen in Fig. 1B implies that the forage diet has a greater diversity of rare OTUs relative to Fig. 1C. This fact can be observed in Table 1, where the ‘long-tailed’ distribution of abundances means that there are more total OTUs observed among the forage-fed animals, despite these animals having many fewer total individuals.

The ecological literature on species richness (the number of OTUs present in our case) and species evenness (whether the numbers of individuals of those species are present in relatively equal numbers) is considerable.^39–41^ However, the exact role of species evenness, in particular, is complex and incompletely understood.^39^ Under some conditions, such as a constant environment, dominance by a few taxa may increase productivity.^42^ However, if the environment is more complex (e.g. certain local regions are more suitable to different taxa, or the environment changes in time), greater evenness of taxa abundance (less dominance) will improve productivity.^42,43^ One can make a plausible argument that the variety and complexity of the nutrients in a forage diet are greater, yielding greater evenness in the OTU abundances. On the other hand, the rumen is a system that has adapted over a long period for forage-like diets, and the differences seen might also be due to this fact. It would be most helpful to develop theories and tests able to distinguish between these two hypotheses.

## Supplementary data

Supplementary data are available at www.dnaresearch.oxfordjournals.org.

## Funding

This work was supported by the USDA National Research Initiative (NRI) (grant 2011-68006-30185).

## Supplementary Material

Supplementary Data
