# Supplementary material for: Diet Alters Both the Structure and Taxonomy of the Ovine Gut Microbial Ecosystem
Source: DNA Res. 2013 Oct 29;21(2):115–25. doi: 10.1093/dnares/dst044 (PMC3989484; doi:10.1093/dnares/dst044)
Supplement: Supplementary Data [file supp_21_2_115__index.html]

Diet Alters Both the Structure and Taxonomy of the Ovine Gut Microbial Ecosystem — Diet Alters Both the Structure and Taxonomy of the Ovine Gut Microbial Ecosystem — Supplementary Data 

# Diet Alters Both the Structure and Taxonomy of the Ovine Gut Microbial Ecosystem

## Supplementary Data

Supplementary Data

**Files in this Data Supplement:**

- Supplementary Data - Pdf file
