# Supplementary material for: Diet Alters Both the Structure and Taxonomy of the Ovine Gut Microbial Ecosystem
Source: DNA Res. 2013 Oct 29;21(2):115–25. doi: 10.1093/dnares/dst044 (PMC3989484; doi:10.1093/dnares/dst044)
Supplement: Supplementary Data [file supp_dst044_dst044supp.pdf]

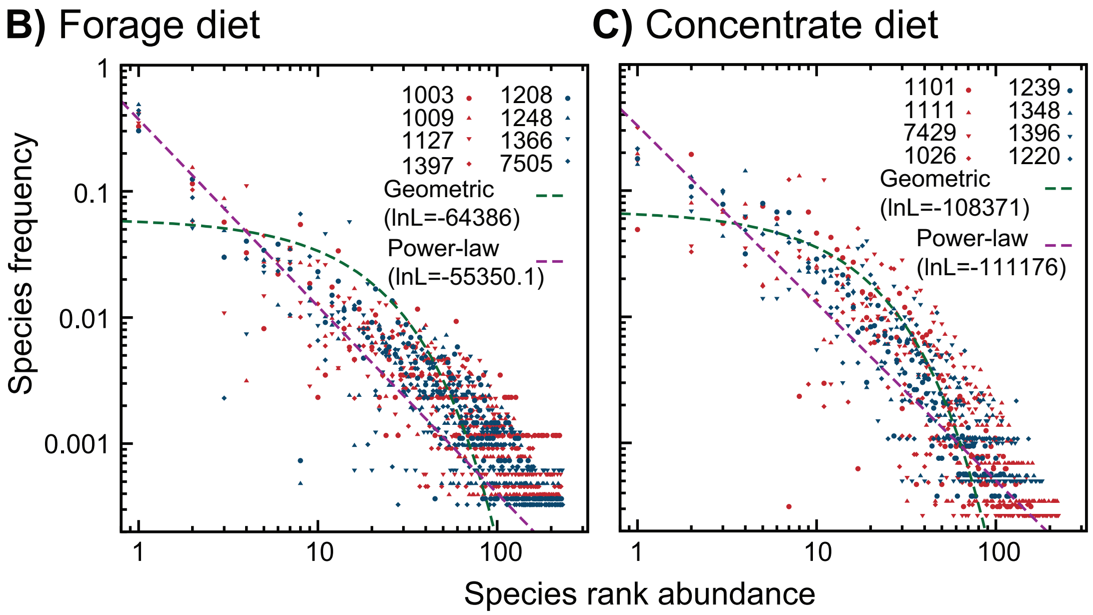

Supplemental Figure 1: Models of the species abundance curves for the two diets including only OTUs derived from the reference database (e.g., 16S_Ref; *Methods*). On the *x*-axis is the rank abundance of each OTU (most abundant OTU is rank 1). On the *y*-axis is the proportion of the total sample for that individual that that rank makes up. The lines describe the fit of a discrete power-law (purple) and a geometric (green; *Methods*) distribution to these data.
